# Epidemiology and prevention of pediatric viral respiratory infections in health-care institutions.

**DOI:** 10.3201/eid0702.010220

**Published:** 2001

**Authors:** D A Goldmann

**Affiliations:** Children's Hospital and Harvard Medical School, Boston, Massachusetts, USA. Goldmann@hub.tch.harvard.edu

## Abstract

Nosocomial viral respiratory infections cause considerable illness and death on pediatric wards. Common causes of these infections include respiratory syncytial virus and influenza. Although primarily a community pathogen, rhinovirus also occasionally results in hospitalization and serious sequelae. This article reviews effective infection control interventions for these three pathogens, as well as ongoing controversies.


### Epidemiology and Prevention of Pediatric Viral Respiratory Infections in Health-Care Institutions

**Donald A. Goldmann**
Children's Hospital and Harvard Medical School, Boston, Massachusetts, USA

Nosocomial viral respiratory infections cause considerable illness and death on pediatric wards. Common causes of these infections include respiratory syncytial virus and influenza. Although primarily a community pathogen, rhinovirus also occasionally results in hospitalization and serious sequelae. This article reviews effective infection control interventions for these three pathogens, as well as ongoing controversies.

Infection control professionals worldwide rely on the Guideline for Isolation Precautions in Hospitals promulgated by the Hospital Infection Control Practices Advisory Committee of the Centers for Disease Control and Prevention (1). This widely venerated document has assumed almost ecclesiastical authority. The guidelines have been framed carefully to reflect current evidence and opinion on the modes of transmission of nosocomial pathogens, and it is this rigorous evidence-based process that insures their credibility. However, scrutiny of guidelines addressing the nosocomial spread of viral pathogens reveals the fragile data on which many of the recommendations are based.

Evidence on modes of transmission of viruses tends to be the most fragmentary and unconvincing. When the first Decennial Conference was held, viral diagnostics was in its infancy, and few hospital clinical laboratories were equipped to assist infection control professionals in understanding the epidemiology of nosocomial viral disease. Moreover, our current knowledge about the spread of infection by droplets and droplet nuclei is a relatively recent phenomenon. It was not that long ago that all infections were thought to be spread by miasms, those putrid vapors emanating from decomposing organic matter and environmental filth. William Farr, an excellent epidemiologist and close colleague of Florence Nightingale, firmly believed that the 1849 cholera outbreak in London was caused by miasms rising from the fetid River Thames. Malaria (literally from the Italian root, *mal aria*, or "bad air") and yellow fever were attributed to miasms before their mosquito vectors were discovered near the turn of the century. Indeed, some authorities predicted with confidence that these diseases, which killed thousands of workers who were trying to dig the Panama Canal, would be eradicated as soon as the canal trench was filled with water, sealing over the miasm-generating tropical ooze. Not until mid-century did Wells et al. at Johns Hopkins demonstrate that tiny droplet nuclei could convey infectious microorganisms over long distances from patient to patient (2).

What, then, do we know about the transmission of common, clinically important nosocomial viruses? Studies of three viruses of importance to pediatric hospital epidemiologists (respiratory syncytial virus [RSV], influenza virus, and rhinovirus) illustrate that modes of transmission have been clarified somewhat but that serious gaps in our knowledge persist. Many of these studies should provide inspiration for young hospital epidemiologists and infection control professionals. Almost without exception, they were performed by hard-nosed investigators who had little, if any, external funding—investigators who exploited serendipitous events or devised and conducted original studies on a shoestring.

### RSV

RSV is the most important cause of respiratory infection in young children worldwide, infecting virtually every child in the first few years of life. Immunity is feeble and fleeting, and repeated infections are the rule. One in every 100 or 200 infected infants requires hospitalization, usually for bronchiolitis. Therefore, pediatric hospital wards are flooded with patients with community-acquired RSV every winter, and failure to follow fastidious infection control procedures inevitably leads to nosocomial transmission (3,4). RSV is, in fact, one of the "perennial weeds" on pediatric wards that Caroline Breese Hall discussed at this same conference 20 years ago (5). The consequences of RSV infection can be especially dire for children with underlying conditions such as prematurity, cardiac and pulmonary disease, or immunosuppression (6-9). Nosocomial RSV infection in immunocompromised adults results in prolonged, substantial illness and even death (10). RSV also takes a heavy toll on members of the nursing and medical staff, with attack rates in some studies approaching 50% (5). Bronchiolitis does not develop in health-care providers because, as adults, they have considerably larger airways than infants; however, severe colds and reactive airway disease do develop (11). Because winter is the busiest time of year on pediatric wards, ill staff members seldom take time off to recuperate, thus serving as efficient vectors in the chain of disease transmission.

Since RSV is a respiratory virus, one might be tempted to speculate that it is transmitted primarily by droplet nuclei or droplet contact. However, Hall et al. demonstrated clearly that contact transmission predominates (12). Freshly infected infants, who were producing copious secretions, were placed in a crib in a room reserved for the study. Volunteers were brought into the room and assigned to one of three

Address for correspondence: Donald A. Goldmann, Division of Infectious Diseases, Children's Hospital, 300 Longwood Avenue, Boston, MA 02115, USA; fax: 617-355-8387; e-mail: Goldmann@hub.tch.harvard.edu groups. "Cuddlers" performed routine care, picked the baby up, and played with the child. "Touchers" had extensive contact with objects in the baby's environment, which had been contaminated heavily with secretions. "Sitters" sat right next to the crib for 3 hours but did not touch anything in the baby's environment. None of the 14 sitters developed RSV infection, but five of the seven cuddlers and four of the 10 touchers became ill.

Infants secrete enormous concentrations of RSV, often more than $10^7$/mL of nasal discharge, and the concentration of virus diminishes only slowly over a period of days (13). Moreover, RSV survives well on fomites; for example, virus can be cultured for >5 hours on impervious surfaces such as bed rails (14). Thus, care givers have numerous opportunities to contaminate their hands during routine care, and unless they wash their hands, virus will be transmitted by indirect contact to other infants. Furthermore, symptomatic infection has a high probability of developing in care givers who touch their eyes or nose with contaminated fingers.

Numerous studies have evaluated potential strategies to control nosocomial transmission of RSV. Gowns and masks were studied before the modes of transmission of RSV were understood fully (15,16). These studies, which were underpowered, did not detect a beneficial impact on the rate of cross-infection. Hall's group, recognizing that the eyes are an unprotected portal for inoculation of virus in health-care workers, evaluated especially designed eye-nose goggles that ward staff could wear when caring for infants infected with RSV (17). Although these goggles reduced the rate of infection in care givers and infants to 5% and 6%, respectively, the goggles were not well accepted by the staff and eventually were abandoned.

Studies at Children's Hospital, Boston, provide considerable support for the key role of contact with contaminated secretions in RSV transmission, as well as the value of wearing gowns and gloves when caring for infected patients (18). Surreptitious surveillance of compliance with gown and glove precautions on a general pediatric ward documented adherence in only 38.5% of encounters with ill infants. When open monitoring, education, and feedback of nosocomial infection rates were introduced, compliance reached levels as high as 95% and remained very good even after surreptitious surveillance was reintroduced. The rate of nosocomial RSV infection fell from 6.4 to 3.1 cases per 1,000 patient days. The magnitude of the effect was by far the greatest at the peak of the winter epidemic in the community, when the ward was crowded with infected infants. Thus, simple barrier precautions, including wearing gloves when touching contaminated objects, proved extremely effective in limiting RSV transmission. Of course, it is possible that excellent compliance with handwashing might obviate the need for gloves, as is the case for all nosocomial infections transmitted from patient to patient by contaminated hands. Isaacs et al. (19) found that handwashing and cohorting were effective in reducing the nosocomial infection rate. For RSV, using a hand antisepsis agent that contains detergent or alcohol is critical. Aqueous chlorhexidine without detergent has poor activity against RSV (20).

Some investigators have advocated performing rapid tests for RSV on all symptomatic infants during the annual RSV season, cohorting RSV-positive patients, and placing them on gown and glove precautions. Madge found that this approach was more effective than gowns and gloves or cohorting alone (21), although compliance was not measured. Snydman noted a reduction in nosocomial infection in a newborn nursery when rapid testing was combined with cohorting, visitation restrictions, and gowns, gloves, and masks (22). However, the cost-effectiveness of routinely testing all symptomatic infants for RSV remains to be demonstrated conclusively. Once the virology laboratory has documented that the RSV season has started, a child with bronchiolitis will likely have RSV, and screening only children who have atypical symptoms may be sufficient.

Recently, investigators using polymerase chain reaction (PCR) to detect RSV RNA suggested that RSV might be transmitted over considerable distances by air (23). RNA was found in air samples taken as far as 7 m from the bedside of infected patients for up to day 7 of hospitalization. However, a positive PCR result does not prove that infectious virus is present, and it seems premature to use such data to refute excellent epidemiologic studies by several groups of investigators documenting the primary importance of contact transmission.

### Influenza

Influenza is a substantial threat to hospitalized patients despite the availability of a relatively effective vaccine and two classes of drugs (M2 ion channel inhibitors and neuraminidase inhibitors) shown to prevent infection in clinical trials (24). Although influenza is widely viewed as affecting primarily elderly patients and adults with coexisting illnesses or conditions, such as chronic pulmonary and cardiac disease, nosocomial transmission has been well documented in young children (25,26). Perhaps nosocomial disease is less frequently diagnosed in hospitalized children because infants are unable to articulate many of influenza's characteristic symptoms, and influenza often presents simply as an episode of fever in this population.

The proper isolation procedures for hospitalized patients with influenza are controversial. Infection can likely be transmitted by direct and indirect contact, as well as by droplet contact. Airborne spread by droplet nuclei has sparked controversy, since true airborne transmission would best be controlled by isolating patients in rooms with negative air pressure and requiring staff to wear masks on entering the room. Such precautions would be costly and difficult to implement at the height of an influenza outbreak.

What is the evidence for airborne transmission of influenza? The explosive nature of influenza outbreaks supports airborne transmission. Some investigators have even suggested that the rapid intercontinental transmission of influenza can be mediated by transport of aerosolized virus on air currents over hundreds to thousands of kilometers in low-pressure centers with frontal waves (27). However, data substantiating the airborne theory of transmission are relatively sparse. Perhaps the most compelling data come from animal models of influenza. Mice inoculated with influenza virus readily transmitted infection to susceptible animals from which they had been separated by double wire screens (28). The attack rate increased at low relative humidity, as would be expected, since virus suspended in aerosolized droplet nuclei survives much longer at lower humidity. Moreover, transmission occurred more frequently when the ventilation in the chamber housing the mice was poor, as Wells established is typical of diseases spread by the airborne route. In a ferret influenza model, infected ferrets transmitted influenza to uninfected ferrets separated by a 9-foot duct with two 90° bends (29). Large droplets certainly would not be able to negotiate such curves, whereas droplet nuclei typically can.

A natural experiment in patients at the Veterans Administration Hospital in Livermore, California, can be viewed as the human counterpart of these animal experiments (30). One building housing 150 patients with tuberculosis and chronic pulmonary disease was ventilated by UV light-irradiated air, whereas another part of the hospital housing 250 tuberculosis patients received nonirradiated air. During the 1957-58 influenza season, the attack rate in patients in the irradiated building (as confirmed serologically) was 2%, but the attack rates among patients and staff in the nonirradiated area were 19% and 18%, respectively.

Probably the most dramatic example of airborne spread in humans occurred during an airplane flight from Anchorage to Kodiak, Alaska (31). At an intermediate stop in Homer, Alaska, the plane had mechanical difficulty and remained on the tarmac for several hours with an inoperative ventilation system. A young woman had boarded the flight in Homer and within 15 minutes developed full-blown symptoms of acute influenza. A point-source outbreak of influenza ensued, and 72% of the 54 passengers became ill within 72 hours. The attack rate was highest in passengers who remained on the crippled plane the longest, and the six passengers who deplaned immediately remained well. Although the passengers who stayed on the plane moved about at will, influenza developed in few of those who had close contact with the index patient.

Since available evidence tends to support airborne transmission of influenza, attempting to place infected patients on precautions suitable for protecting susceptible patients and staff from virus-laden droplet nuclei seems prudent. Of course, improved compliance with current recommendations for immunizing health-care workers remains the key to influenza control in the hospital. Most facilities will be severely challenged if they try to isolate all patients with symptoms compatible with influenza.

### Rhinovirus

Although nosocomial rhinovirus infection is not as substantial a problem as RSV and influenza on pediatric wards, it can have serious sequelae in premature neonates and children with chronic diseases or immunosuppression (32). For example, in another session at this decennial meeting, Huskins and his colleagues at Children's Hospital, Boston, report an outbreak of rhinovirus infection at a pediatric chronic-care facility that was associated with considerable illness and death. However, there is another reason to discuss the transmission of rhinovirus–namely, that this pathogen demonstrates the difficulty in proving conclusively how respiratory viruses are transmitted.

The common cold is a profound nuisance in everyday life, although seldom a cause of serious illness. The average child can expect to have four to eight episodes per year, and adults three to five infections. Many viruses, such as parainfluenza, RSV, and coronavirus, can produce similar symptoms, but rhinovirus is by far the most frequent etiologic agent. Repeated colds are virtually guaranteed because there are >100 distinct rhinovirus serotypes, and infection with one serotype does not confer substantial immunity against the others.

A prodigious volume of work at the Common Cold Research Unit in Salisbury, England, following World War II established that colds could be produced by inoculating secretions into the nose or eye of volunteers (33). These rather crude experiments were replicated with nasal inoculation of small concentrations of rhinovirus once the specific viral agents that cause the common cold were elucidated (34). Presumably, therefore, persons might acquire rhinovirus by touching their nasal or ocular mucosa with contaminated fingers. A study by Hendley et al. at the University of Virginia demonstrated that health-care workers are not immune to practices that might promote self-inoculation (35). One third of grand-rounds attendees picked their nose, and one in 2.7 rubbed their eyes during a 1-hour lecture. Subsequent work demonstrated that it was difficult to transmit rhinovirus by kissing (36), and that exposure to cold did not increase the likelihood of "catching a cold" (37).

These studies could not answer the central question of whether rhinovirus is transmitted primarily by direct contact, indirect contact, droplet contact, or droplet nuclei. Unfortunately, considerable additional investigation has not resolved the issue completely (38). Essentially, two experimental approaches, both highly contrived, have come to different conclusions. Work by Hendley and Gwaltney at the University of Virginia generally has supported transmission by hand contact and self-inoculation, while experiments by Dick at the University of Wisconsin have favored spread by large droplets, droplet nuclei, or both.

The Virginia group demonstrated that adults with experimental rhinovirus colds readily contaminated their hands and that rhinovirus could be recovered from 43% of plastic tiles they touched with their contaminated fingers (39). Adults with natural rhinovirus colds contaminated their hands in 39% of cases, and virus was found on 6% of objects in their homes (35,40). Virus could survive from a few hours to as long as 4 days on nonporous surfaces, and for at least 2 hours on human skin (35). Volunteers who had contact with contaminated objects or with fingers of persons with rhinovirus colds had a high rate of infection when they intentionally touched their eyes or nose. Infection generally could be prevented by treating contaminated surfaces with disinfectant or applying iodine to fingers (39).

In a labor-intensive, randomized clinical trial, the Virginia group found that treating mothers' fingers with iodine reduced the rate of secondary infection (38). Specifically, as soon as a cold occurred in another member of the family, mothers were instructed to dip their fingers in iodine or placebo when they awoke in the morning, every 3 to 4 hours during the day, and after activities that might wash the iodine from the skin. The investigators counted on the well-established residual activity of iodine to kill virus on contact. Over the 4-year study period, the secondary attack rate for colds in the intervention group was 7%, versus 20% in the control group. In the iodine-treated group, no confirmed rhinovirus infection occurred in susceptible mothers who had been exposed to 11 index cases. In contrast, five infections occurred after 16 exposures in the placebo group, although this difference was not significant.

These studies provide considerable evidence for indirect contact transmission by contaminated fomites and fingers. In other experiments, the Virginia investigators found little support for transmission via large respiratory droplets or droplet nuclei. Exposure of susceptible volunteers to highly symptomatic volunteers across a small table (droplet contact and droplet nucleus transmission) or a double-wire barrier (droplet nucleus spread) resulted in infections in 1 of 12 and zero of 10 subjects, respectively (39). These rates of transmission were far less than the 11 infections among 15 persons (73%) who self-inoculated their mucous membranes with contaminated fingers.

Meanwhile, the Wisconsin group was developing models to study transmission of rhinovirus colds, building on observations showing high attack rates among men crowded together in a small hut in Antarctica (41). In one such model, symptomatic volunteers were housed with susceptible volunteers in a room approximately 12-by-6-by-3 m (42). The subjects played various board, card, and video games during the study period. Since viral titers in nasal secretions fall as symptoms diminish, volunteers were replaced with highly symptomatic persons as soon as they experienced reduced rhinorrhea or sneezing. The average length of exposure required for transmission was very high, 200 hours of exposure to achieve a 50% attack rate. Based on these results, Dick et al. suggest that exposure times in the Virginia studies were too short to exclude droplet and airborne transmission.

In additional experiments, the Wisconsin group extended these studies by having volunteers play poker for 12 hours while sitting at round tables (43). Three experiments were performed involving 24 symptomatic "donors" and 36 susceptible "recipients." Half of the recipients were fitted with restraints, either arm braces that allowed them to reach their cards but not touch their face, or a plastic shield that left their hands free but did not allow them to reach their eyes or nose. Despite these barriers, the attack rates were 56% and 67%, respectively, strongly favoring transmission by air since self-inoculation was impossible. Moreover, when 12 additional susceptible volunteers were brought to a separate room to play poker with chips and cards that were literally soaked with contaminated secretions from donors, no rhinovirus infections occurred. In addition, little virus was found on the chips and cards. The Wisconsin group suggested that the relatively high attack rates seen in the self-inoculation studies conducted by the Virginia group might be attributable to intensive exposure to fresh wet secretions (e.g., the volunteers literally blew their noses into their hands).

The above studies provide only a glimpse of the extensive literature on the transmission of rhinovirus colds, but controversy still simmers. The prudent person probably will wash his or her hands after shaking hands with someone who has a cold or after touching environmental objects potentially contaminated with relatively fresh secretions. Alcohol-based, waterless antiseptics are ideal for this purpose. Although droplet contact or airborne transmission of rhinovirus infection is possible, prolonged and close exposure is apparently required.

Dr. Goldmann, who is professor of pediatrics at Harvard Medical School, has a research focus on the epidemiology and control of hospital-acquired infections, especially antimicrobial drug-resistant infections in intensive care units. In addition, he studies the epidemiology and prevention of medical errors and adverse events in pediatrics. Dr. Goldmann collaborates with colleagues at the Channing Laboratory in Boston regarding the pathogenesis of staphylococcal foreign body infections and is working to develop imunologic approaches for their prevention.

### References

1. Garner JS, for the Hospital Infection Control Practices Advisory Committee. Infect Control Hosp Epidemiol 1996;17:53-80.
2. Wells WF. Airborne contagion and air hygiene. Boston: Harvard University Press; 1955.
3. Hall CB, Geiman JM, Douglas RG, Jr, Meagher MP. Control of nosocomial respiratory syncytial viral infections. Pediatrics 1978;62:728-32.
4. Hall CB, Douglas RG, Jr, Geiman JM, Messner MK. Nosocomial respiratory syncytial virus infection. N Engl J Med 1975;293:1343-6.
5. Hall CB. Nosocomial viral respiratory infections: Perennial weeds on pediatric wards. Am J Med 1981;70:670-6.
6. Hall CB, Kopelman AE, Douglas RG Jr, Geiman JM, Meagher MP. Neonatal respiratory syncytial virus infection. N Engl J Med 1979;300:393-6.
7. Ogra PL, Patel J. Respiratory syncytial virus infection and the immunocompromised host. Pediatr Infect Dis J 1988;7:246-9.
8. Hall CB, Powell KR, MacDonald NE, Gala CL, Menegus ME, Suffin SC, et al. Respiratory syncytial viral infection in children with compromised immune function. N Engl J Med 1986;315:77-81.
9. MacDonald NE, Hall CB, Suffin SC, Alexson C, Harris PJ, Manning JA. Respiratory syncytial viral infection in infants with congenital heart disease. N Engl J Med 1982;307:397-400.
10. Englund JA, Anderson LJ, Rhame FS. Nosocomial transmission of respiratory syncytial virus in immunocompromised adults. J Clin Microbiol 1991;29:115-19.
11. Hall WJ, Hall CB, Speers DM. Respiratory syncytial virus infection in adults: Clinical, virologic, and serial pulmonary function studies. Ann Intern Med 1978;88:203-5.
12. Hall CB, Douglas RG Jr. Modes of transmission of respiratory syncytial virus. J Pediatr 1981;99:100-3.
13. Hall CB, Douglas RG Jr, Geiman JM. Respiratory syncytial virus infections in infants: Quantitation and duration of shedding. J Pediatr 1976;89:11-15.
14. Hall CB, Douglas RG, Jr, Geiman JM. Possible transmission by fomites of respiratory syncytial virus. J Infect Dis 1978:88:203-5.
15. Murphy D, Todd JK, Chao RK, Orr I, McIntosh K. The use of gowns and masks to control respiratory illness in pediatric hospital personnel. J Pediatr 1981;99:746-50.
16. Hall CB, Douglas RG Jr. Nosocomial respiratory syncytial viral infection. Am J Dis Child 1981;135:512-15.
17. Gala CL, Hall CB, Schnabel KC, Pincus PH, Blossom P, Hidreth SW, et al. The use of eye-nose goggles to control nosocomial respiratory syncytial virus infection. JAMA 1986;19:2706-8.
18. Leclair JM, Freeman J, Sullivan BF, Crowley CM, Goldmann DA. Prevention of nosocomial respiratory virus infections through compliance with glove and gown isolation precautions. N Engl J Med 1987;317:329-34.
19. Isaacs D, Dickson H, O'Callaghan C, Sheaves R, Winter A, Moxon ER. Handwashing and cohorting in prevention of hospital acquired infections with respiratory syncytial virus. Arch Dis Child 1991;66:227-31.
20. Platt J, Bucknall RA. The disinfection of respiratory syncytial virus by isopropanol and a chlorhexidine-detergent handwash. J Hosp Infect 1985;6:89-94.
21. Madge P, Paton JY, McColl JH, Mackie PLK. Prospective controlled study of four infection control procedures to prevent nosocomial infection with respiratory syncytial virus. Lancet 1992;340:1079-83.
22. Snydman DR, Greer C, Meissner HC, McIntosh K. Prevention of nosocomial transmission of respiratory syncytial virus in a newborn nursery. Infect Control Hosp Epidemiol 1988;9:105-8.

23. Aintablian N, Walpita P, Sawyer MH. Detection of *Bordetella pertussis* and respiratory syncytial virus in air samples from hospital rooms. Infect Control Hosp Epidemiol 1998;12:918-23.
24. Wenzel RP. Expanding the treatment options for influenza. JAMA 2000;283:1057-9.
25. Gardner PS, Court SDM, Brocklebank JT, Downham MA, Weightman D. Virus cross-infection in pediatric wards. Br Med J 1973;2:571-5.
26. Hall CB, Douglas RG Jr. Nosocomial influenza infection as a cause of intercurrent fevers in infants. Pediatrics 1975;55:673-6.
27. Hammond GW, Raddatz RL, Gelskey DE. Impact of atmospheric dispersion and transport of viral aerosols on the epidemiology of influenza. Rev Infect Dis 1989;11:494-7.
28. Schulman JL, Kilbourne ED. Airborne transmission of influenza virus infection in mice. Nature 1962;195:1129-30.
29. Andrewes CH, Glover RE. Spread of infection from the respiratory tract of the ferret. I. Transmission of influenza A virus. Br J Exp Pathol 1941;22:91-7.
30. McLean RL. General discussion. Am Rev Respir Dis 1961;83:36-8.
31. Moser MR, Bender TR, Margolis HS, Noble GR, Kendal AP, Ritter DG. An outbreak of influenza aboard a commercial airliner. Am J Epidemiol 1979;110:1-6.
32. Valenti WM, Clarke TA, Hall CB, Menegus MA, Shapiro DL. Concurrent outbreaks of rhinovirus and respiratory syncytial virus in an intensive care nursery: Epidemiology and associated risk factors. J Pediatr 1982;100:722-6.
33. Tyrell DAJ. Common colds and related diseases. Baltimore:Williams and Wilkins; 1965.
34. Douglas RG Jr. Pathogenesis of rhinovirus colds in human volunteers. Ann Otol Rhinol Laryngol 1970;79:563-71.
35. Hendley JO, Wenzel RP, Gwaltney JM Jr. Transmission of rhinovirus colds by self-inoculation. N Engl J Med 1973;288:1361-4.
36. D'Alessio DJ, Peterson JA, Dick CR, Dick EC. Transmission of experimental rhinovirus colds in volunteer married couples. J Infect Dis 1976;133:28-36.
37. Douglas RG Jr, Lindgren KM, Couch RB. Exposure to cold environment and rhinovirus common cold: Failure to demonstrate effect. N Engl J Med 1968;279:742-7.
38. Hendley JO, Gwaltney JM Jr. Mechanisms of transmission of rhinovirus infections. Epidemiol Rev 1988;10:242-58.
39. Gwaltney JM Jr, Hendley JO. Transmission of experimental rhinovirus infection by contaminated surfaces. Am J Epidemiol 1982;116:828-33.
40. Gwaltney JM Jr, Moskalski PB, Hendley JO. Hand-to-hand transmission of rhinovirus colds. Ann Intern Med 1978;88:463-7.
41. Holmes J, Reed S, Stott E, Tyrrell D. Studies of experimental rhinovirus type 2 infections in polar isolation and in England. J Hyg Camb 1976;76:379-3.
42. Jennings LC, Dick EC. Transmission and control of rhinovirus colds. Eur J Epidemiol 1987;3:327-35.
43. Dick EC, Jennings LC, Mink KA, Wartgow CD, Inhorn SL. Aerosol transmission of rhinovirus colds. J Infect Dis 1987;156:442-8.
